# Siberian larch (*Larix sibirica* Ledeb.) mitochondrial genome assembled using both short and long nucleotide sequence reads is currently the largest known mitogenome

**DOI:** 10.1186/s12864-020-07061-4

**Published:** 2020-09-23

**Authors:** Yuliya A. Putintseva, Eugeniya I. Bondar, Evgeniy P. Simonov, Vadim V. Sharov, Natalya V. Oreshkova, Dmitry A. Kuzmin, Yuri M. Konstantinov, Vladimir N. Shmakov, Vadim I. Belkov, Michael G. Sadovsky, Olivier Keech, Konstantin V. Krutovsky

**Affiliations:** 1 Laboratory of Forest Genomics, Genome Research and Education Center, Institute of Fundamental Biology and Biotechnology, Siberian Federal University, Krasnoyarsk, 660036 Russia; 2 Laboratory of Genomic Research and Biotechnology, Federal Research Center “Krasnoyarsk Science Center”, Siberian Branch, Russian Academy of Sciences, Krasnoyarsk, 660036 Russia; 3 Institute of Environmental and Agricultural Biology (X-BIO), University of Tyumen, Tyumen, 625003 Russia; 4 Department of High Performance Computing, Institute of Space and Information Technologies, Siberian Federal University, Krasnoyarsk, 660074 Russia; 5 Laboratory of Forest Genetics and Selection, V. N. Sukachev Institute of Forest, Siberian Branch, Russian Academy of Sciences, Krasnoyarsk, 660036 Russia; 6 Laboratory of Plant Genetic Engineering, Siberian Institute of Plant Physiology and Biochemistry, Siberian Branch, Russian Academy of Sciences, Irkutsk, 664033 Russia; 7 Institute of Computational Modeling, Siberian Branch, Russian Academy of Sciences, Krasnoyarsk, 660036 Russia; 8 Department of Plant Physiology, UPSC, Umeå University, S-90187 Umeå, Sweden; 9 Department of Forest Genetics and Forest Tree Breeding, Georg-August University of Göttingen, 37077 Göttingen, Germany; 10 Center for Integrated Breeding Research, George-August University of Göttingen, 37075 Göttingen, Germany; 11 Laboratory of Population Genetics, N.I. Vavilov Institute of General Genetics, Russian Academy of Sciences, Moscow, 119333 Russia; 12 Department of Ecosystem Science and Management, Texas A&M University, College Station, TX 77843-2138 USA

**Keywords:** *Larix sibirica*, Long reads, Mitochondrial genome, mtDNA, Nucleotide sequence, RNA editing

## Abstract

**Background:**

Plant mitochondrial genomes (mitogenomes) can be structurally complex while their size can vary from ~ 222 Kbp in *Brassica napus* to 11.3 Mbp in *Silene conica*. To date, in comparison with the number of plant species, only a few plant mitogenomes have been sequenced and released, particularly for conifers (the Pinaceae family). Conifers cover an ancient group of land plants that includes about 600 species, and which are of great ecological and economical value. Among them, Siberian larch (*Larix sibirica* Ledeb.) represents one of the keystone species in Siberian boreal forests. Yet, despite its importance for evolutionary and population studies, the mitogenome of Siberian larch has not yet been assembled and studied.

**Results:**

Two sources of DNA sequences were used to search for mitochondrial DNA (mtDNA) sequences: mtDNA enriched samples and nucleotide reads generated in the de novo whole genome sequencing project, respectively. The assembly of the Siberian larch mitogenome contained nine contigs, with the shortest and the largest contigs being 24,767 bp and 4,008,762 bp, respectively. The total size of the genome was estimated at 11.7 Mbp. In total, 40 protein-coding, 34 tRNA, and 3 rRNA genes and numerous repetitive elements (REs) were annotated in this mitogenome. In total, 864 C-to-U RNA editing sites were found for 38 out of 40 protein-coding genes. The immense size of this genome, currently the largest reported, can be partly explained by variable numbers of mobile genetic elements, and introns, but unlikely by plasmid-related sequences. We found few plasmid-like insertions representing only 0.11% of the entire Siberian larch mitogenome.

**Conclusions:**

Our study showed that the size of the Siberian larch mitogenome is much larger than in other so far studied Gymnosperms, and in the same range as for the annual flowering plant *Silene conica* (11.3 Mbp). Similar to other species, the Siberian larch mitogenome contains relatively few genes, and despite its huge size, the repeated and low complexity regions cover only 14.46% of the mitogenome sequence.

## Background

Since the first plant mitogenome of the common liverwort has been sequenced [1], not as many mitogenomes were sequenced and assembled in plants as in animals. Although more than 9000 complete mitogenomes have been deposited in NCBI Genbank (July 2020), only 235 of them belong to plants (https://www.ncbi.nlm.nih.gov/genome), and the vast majority of these mitogenomes are from angiosperms. To date, the complete assembly and accurate annotation of plant mitogenomes remain challenging due to their complex and often perplexing structure.

Mitogenome size in seed plants can vary by at least one order of magnitude ranging from ~ 222 Kbp in *Brassica napus* [2] and ~ 316 Kbp in *Allium cepa* [3] to ~ 3.9 Mbp in *Amborella trichopoda* [4] and a striking ~ 11.3 Mbp in *Silene conica* [5]. Such dispersion may be explained by the abundance of noncoding and repeated elements [6]. In contrast to the relatively compact and tightly packed genomes of most animal mitochondria (~ 15–20 Kbp) [7], plant mitogenomes are enriched with introns, intergenic sequences, repetitive and mobile elements [8], and show a wide diversity in gene content and genomic architecture [9, 10], although coding sequences are relatively conserved in the core mitochondrial genes [11–13].

Both plant and animal mitogenomes were used to be considered as a single circular chromosome, and until recently, this model predominated in mitogenome assembly strategies. Today, however, substantial evidence has been accumulating in support of the notion that these genomes possess more complex and multifarious structure. For several plant species, the multi-chromosomal structure of mitogenome has been shown [14]. For instance, the mitogenome of cucumber *Cucumis sativus* can be assembled only into three circular chromosomes of 1.6 Mbp, 84 Kbp, and 45 Kbp in size, respectively [15], and the mitogenome of *Silene noctiflora* (6.7 Mbp) is fragmented into more than 50 circular chromosomes [5]. Interestingly, mitogenomes of numerous vascular plants exhibit presence of alien sequences received either by means of intracellular gene transfer from plastid [16, 17] or horizontal gene transfer from mitochondria of other species [18]. Most of these foreign sequences are noncoding or pseudogenes, though sometimes they occur in coding regions [17]. Conversely, gene transfer from mitogenome to plastid is considered to be rare and has been reported only in a few cases [19–22]. Moreover, genes of plant mitochondria are commonly considered to evolve more slowly than plastidial or nuclear ones, while the mutation rate in coding regions of plant mitogenomes is about two orders of magnitude lower than in genes of animal mitochondria [23–25]. With that being said, it has been reported that the rate at which plant mitogenomes accumulate substitutions can substantially vary both between genes and species [26, 27].

The utility of mitogenome sequences as a source of genetic markers has been extensively documented and is thus unquestionable [28, 29]. Many mitochondrial genes such as *atp1*, *cob*, *cox1*, *cox2*, and *cox3* are widely used to resolve phylogenetic relationships between lineages, conduct biodiversity analyses and construct phylogeographic and evolutionary history of species. Furthermore, the maternal inheritance of mitochondria in plants [30] as well as the large number of copies per cell strengthened their use for various applications [31].

The number of evolutionary and systematic studies based on comparative analysis of complete plant mitogenome sequences has significantly increased in the last decade, notably due to the advent of better sequencing methods. However, there are still very little published comparative mitogenome studies in gymnosperms, one of the oldest among the major plant clades comprising 14 families with more than 1000 species. Among all plant mitochondria genomes available, few gymnosperm mitogenomes have been fully assembled and properly annotated: *Cycas taitungensis* (415 Kbp) [32], a ginkgo tree *Ginkgo biloba* (347 Kbp) [33], a gnetophyte *Welwitschia mirabilis* (979 Kbp) [33], the Japanese *yew Taxus cuspidata* (469 Kbp) [34] and six conifer species (the Pinaceae family), such as white spruce – *Picea glauca* (5.9 Mbp) [35], Norway spruce – *Picea abies* (4.3 Mbp [36] or 4.9 Mbp [37]), loblolly pine – *Pinus taeda* (1.25 Mbp [38] or 1.19 Mbp [https://www.ncbi.nlm.nih.gov/nuccore/NC_039746.1/]), sugar pine – *Pinus lambertiana* (3.9 Mbp) [39], Scots pine – *Pinus sylvestris* (986 Kbp [https://www.ncbi.nlm.nih.gov/assembly/GCA_900143225.1/]), and Siberian larch – *Larix sibirica* (11.7 Mbp) presented here.

In this study, we report on the sequencing, assembly, and annotation of the mitogenome of Siberian larch, a key species for the Siberian boreal forest and ecosystem. Furthermore, several methods were employed to verify and evaluate the correctness of the assemblies.

## Results

### Sequencing and preliminary assembly using short paired-end (PE) and mate-pair (MP) Illumina reads

Based on ~ 19.7 million of paired-end (PE) nucleotide reads produced by Illumina HiSeq 2000 using mitochondrial DNA (mtDNA) enriched samples and ~ 625.5 million of mate-pair (MP) nucleotide reads produced by Illumina HiSeq 2000 for the whole nuclear genome assembly [40] a preliminary draft assembly with total length of 545 Mbp was generated using the CLC Assembly Cell and the BESST software. After mapping the draft assembly contigs to mitogenomes of seed plants and filtering out contamination with nuclear and plastid contigs, the Siberian larch mitogenome was assembled into 53 scaffolds with total length of 11.09 Mbp.

### Final hybrid re-assembly using both long MinION and short paired-end (PE) Illumina reads

To improve the assembly of the Siberian larch mitogenome, we used the same PE reads as in the preliminary CLC assembly generated by Illumina HiSeq 2000 and long reads generated by Oxford Nanopore MinION and MaSuRCa v.3.2.8 pipeline. MP reads were used only for the preliminary CLC assembly, but not for the MaSuRCa assembly. After mapping the MaSuRCa assembly to plant mitochondrial database, we finally assembled nine mitochondrial contigs resulting in a total length of 11.7 Mbp. The largest contig was 4,008,762 bp long.

To further evaluate the accuracy of the final assembly, we ran REAPR v1.0.18 [41], which reported 92.13% of error free bases in the Siberian larch mitogenome assembly (Additional file 1). It is comparable to 86% for the GRCh37 human reference genome or 90.3% for *C. elegans* genome.

### Repetitive elements

**Fig. 1:**
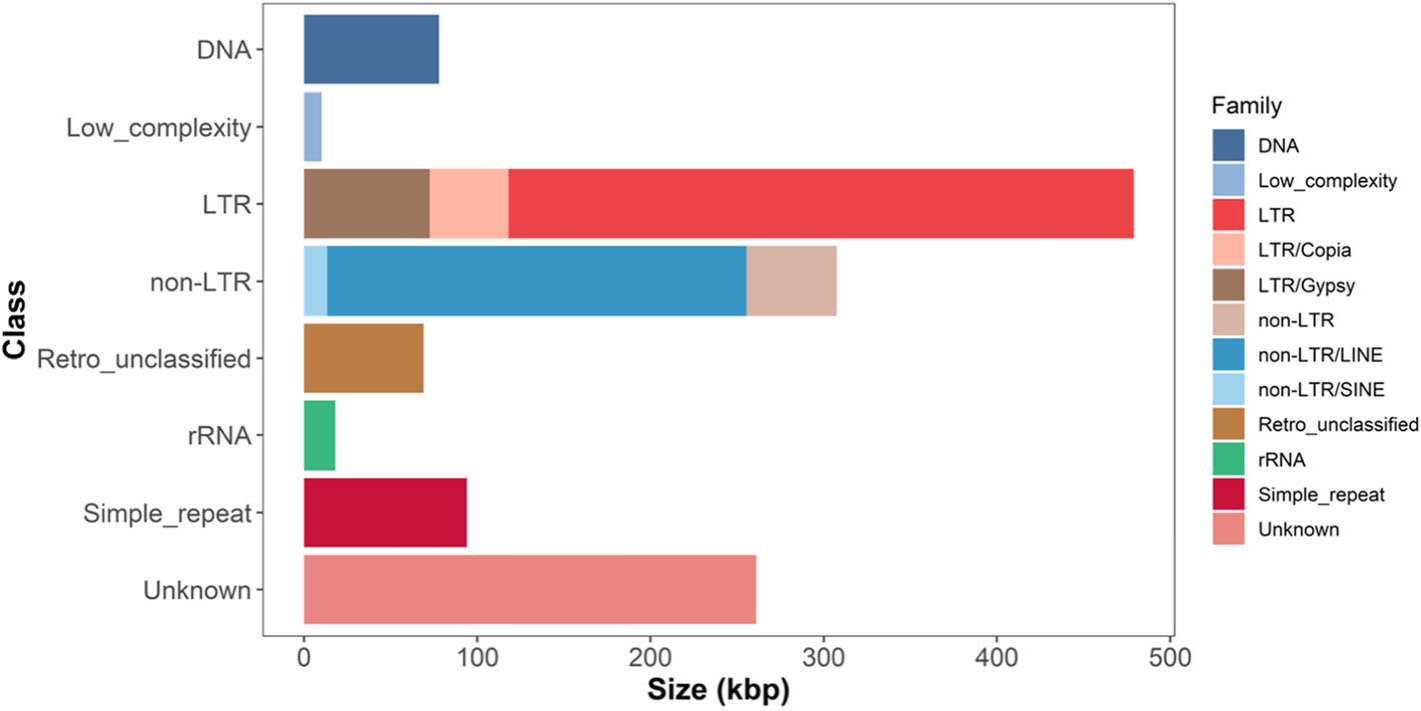
Summary of repetitive elements identified in Siberian larch mitogenome

We used RepeatModeler and TEclass to discover and classify highly repetitive elements (REs) in the assembled contigs, respectively. RepeatModeler found 122 mobile elements, among which TEclass identified 27 DNA transposons, 38 long terminal repeats (LTRs), 12 long interspersed nuclear elements (*LINEs*), and 9 short interspersed nuclear elements (*SINEs*). In total, 7691 REs were identified using a combined repeat library, which represent 11.14% of the 11.7 Mbp mitogenome assembly. LTRs, *LINEs*, simple repeats, and class II DNA transposons were the most abundant among classified repeats (4.05, 2.05, 0.80, and 0.66%, respectively; Fig. 1, Table 1). In total, interspersed repeats composed 7.9% of Siberian larch mitogenome.

**Table 1:** Repetitive elements in the Siberian larch mitogenome annotated using RepeatMasker, TEclass, and RepeatModeler with RepBase, MIPS-REdat, CPRD, PIER v1.0, and de novo libraries

Type	Group	Superfamily (Clade)	Length, bp	%
Class I	LTR retrotransposon	*Copia*	45,496	0.385
		*DIRS*	1745	0.015
		*Gymny*	2085	0.018
		*Gypsy*	70,511	0.597
		Other	359,085	3.039
		Total	478,922	4.054
	non-LTR retrotransposon	*LINE*	241,900	2.047
		*SINE*	13,436	0.114
		*Penelope*	983	0.008
		*I*	48,868	0.414
		Other	2547	0.022
		Total	307,734	2.605
	Unclassified retrotransposon		69,015	0.584
Class II	DNA transposons	*EnSpm*	509	0.004
		*Harbinger*	50	0.0004
		*hAT*	1525	0.013
		*Helitron*	1054	0.009
		*Maverick* (*Polinton*)	18	0.0001
		*MuDR*	57	0.0002
		*TcMar*	78	0.001
		*TIR*	3459	0.029
		Other	71,174	0.602
		Total	77,924	0.658
Low complexity regions			10,177	0.086
rRNA			18,009	0.152
Simple repeats			93,903	0.795
Unknown			261,098	2.210
Grand Total			1,316,782	11.144

LTRs were represented mainly by *Gypsy*, *Copia*, *DIRS*, and *Gymny* families. The latter was previously found in pine genome and is related to *Athila* elements in *Arabidopsis* [42]. Among non-LTR retrotransposons *SINE*, *I*, and *Penelope* together comprise about 20.56% of the non-LTRs, which is 0.53% of the entire mitogenome. RepeatMasker found three *Penelope* elements, all with relatively high score and length between 57 and 813 bp. The *Penelope*-like elements are considered to be common in animals but were also found recently in conifers, particularly in loblolly pine genome [43]. It was proposed that these elements were transferred to a conifers’ ancestor approximately 340 mya [43].

Class II DNA transposons composed 0.66% of the mitogenome assembly and were represented by *EnSpm*, *Harbinger*, *hAT*, *Helitron*, *MuDR*, *TcMar*, and *Maverick* (*Polinton*) repeat families (Table 1). Only one 18 bp long *Polinton* element was found in the mitogenome assembly. Although *Polintons* are not typical for plant genomes, some *Maverick*-related elements were found in the cytoplasm and mitochondria of some plants and fungi [44, 45].

### Search for plasmids

Only a small 42 bp long insert matching plasmid sequence identified in mitochondria of *Picea abies* (L.) Karst (NCBI GenBank accession number AJ225562) was found in contig 1 in the 608,296–608,337 position, yet with 100% identity (Table 2). Among 891 broad host range plasmids (NCBI GenBank taxid:36549), no plasmid-like inserts were identified in the contigs using megablast search for highly similar sequences, and only a few rather short plasmid-like inserts were identified by discontiguous megablast used to search for more dissimilar sequences and by blastn used to search for somewhat similar sequences counting in total for 730 bp and 12,940 bp, respectively (Table 2). In total, considering somewhat similar sequences, plasmid-like insertions represented only 0.11% of the entire mitogenome.

**Table 2:** Plasmid-like inserts in the Siberian larch mitogenome

Contig		***Picea abies*** ^**a**^		Broad host range plasmids (NCBI GenBank taxid:36549)					
#	length, bp	N	A	highly similar sequences (megablast)	more dissimilar sequences (discontiguous megablast)		somewhat similar sequences (blastn)		
				N	N^**c**^	A	N^**d**^	A	% of total
1	4,008,762	1^**b**^	42	none	2	206	50	2038	0.05
2	3,031,766	none	–	none	3	269	66	2387	0.08
3	2,293,716	none	–	none	none	–	69	2397	0.10
4	1,382,651	none	–	none	none	–	52	1946	0.14
5	469,276	none	–	none	none	–	17	463	0.10
6	204,181	none	–	none	none	–	51	1829	0.90
7	142,269	none	–	none	2	206	16	481	0.34
8	105,151	none	–	none	none	–	34	1107	1.05
9	24,767	none	–	none	1	49	11	292	1.18
Total	11,662,539	1	42	0	8	730	366	12,940	0.11

N - number of matching sites; A - alignment length, bp

^a^*Picea abies* mitochondrial plasmid-like DNA (NCBI GenBank accession number AJ225562)

^b^one short site 608,296–608,337 with identity 100%

^c^contig 1: two sites 2,426,685–2,426,787 and 724,520–724,622, both matching Z34929 with identity 78%; contig 2: three sites 1,294,914–1,295,016 and 865,049–865,151, both matching Z34929 with identity 78%, and 738,551–738,613 matching CP021634 with identity 78%; contig 7: two sites 127,403–127,505 and 37,625–37,727, both matching Z34929 with identity 78%; contig 9: one site 4507–4555 (84%, CP021611)

^d^Data on plasmids matching inserts in contigs and their NCBI GenBank accession numbers are provided in Additional file 2

### Genome annotation

Annotation of the Siberian larch mitogenome identified a set of rRNA, tRNA, and protein-coding genes that are typical for gymnosperms: *atp1*, *atp4*, *atp6*, *atp8, atp9*, *ccmB*, *ccmC*, *ccmFc*, *ccmFn, cob, cox1, cox2, cox3, matR, mttB, nad1, nad2, nad3, nad4, nad4L, nad5, nad6, nad7, nad9, rpl2, rpl5, rpl16, rps1, rps2, rps3, rps4, rps7, rps10, rps11, rps12, rps13, rps14, rps19, sdh3,* and *sdh4*. Two copies of *atp8* were found.

Plastid-derived DNA sequences (MTPTs) were searched by comparing the mtDNA against the chloroplast DNA (cpDNA) of Siberian larch [46]. Twenty MTPTs with total length of 43,951 bp were found in five mitochondrial contigs (Table 3).

**Table 3:** MTPT in the Siberian larch mitogenome

Mitochondrial contig	Alignment length (mitochondrial/chloroplast), bp	Plastid gene
contig 1	291/290	*rrn23*
contig 1	3164/3165	*ycf2*
contig 1	1881/1881	*trnV*
contig 1	1631/1631	*atpB*
contig 1	459/459	*rbcL*
contig 2	7245/7245	*trnN, chlL, chlN, ycf1*
contig 2	1486/1485	noncoding plastid fragment
contig 2	298/304	*chlB*
contig 2	3495/3495	*accD, trnR, psaI, ycf4*
contig 2	252/252	*psbE*
contig 2	3104/3104	*rps12, trnW, trnP, psaJ, rpl33, rps18, rpl20*
contig 3	442/390	*rpoB*
contig 3	10,023/9995	*trnL, trnT, rps4, trnS, ycf3, psaA, psaB*
contig 3	452/436	*psbC, psbD*
contig 3	686/670	noncoding plastid fragment
contig 4	173/173	*atpA*
contig 4	1502/1514	*rpl2*
contig 4	649/705	*trnI*
contig 4	1907/1908	*rps12, rps7*
contig 8	4588/4588	*petB, petD, rpoA, rps11, rpl36, infA*

### RNA sequencing and C-to-U RNA editing

The transcriptome data used to verify RNA editing sites are provided in Table 4.

**Table 4:** The RNA-seq and transcriptome data used to verify RNA editing sites

Total number of reads	11,481,272
**Read length, bp**	35–142 (mean = 95.6)
**Total transcriptome length, bp**	27,060,589
**Total Trinity ‘genes’**	42,734
**Total Trinity transcripts**	46,618
**GC, %**	43.47
**Max contig length, bp**	10,795
**Contig N50, bp**	790
**Contig N90, bp**	260
**Median contig length, bp**	361
**Average contig length, bp**	580.5

Among 438 RNA editing sites predicted by the PREP-Mt program almost 90% were also verified by aligning RNA-seq reads to the annotated mitochondrial genes and then by calling variants using the CLC Genomic Workbench. Many additional sites were found only by variant-calling. They were likely specific for Siberian larch and included in the final annotation. In total, 864 C-to-U RNA editing sites were found for 38 among 40 protein-coding genes. The RNA editing was not found only for two genes, *nad1* and *nad4*. Maximum number of the editing sites (80) was observed for the *nad5* gene. Editing the RNA helped us identify start codons in *atp6*, *rps3*, *rps10*, *rps19*, and *mttB* genes and stop codons in *nad4L*, *sdh3*, and *atp9* genes.

## Discussion

The whole genome sequencing data were used in the previous studies of conifer mitogenomes, while sequencing and assembly of *Cycas*, *Ginkgo*, and *Welwitschia* mitogenomes were carried out specifically using enriched mtDNA. Our study benefits from both approaches by simply having more mitochondrial reads, but mtDNA enrichment was not as critical as having long reads.

Almost all studies that used whole genome sequencing data followed similar strategies to assemble and verify putative mitochondrial sequences. They separated mitochondrial sequences from nuclear and chloroplast by their length, depth of coverage, and GC content [35, 36, 38], additionally checking for contamination by aligning obtained sequences either to the NCBI GenBank nucleotide (nt) database [37] or to custom database of plant mitogenomes [38]. Annotation process for abovementioned species included BLAST search against database of homologous genes from available plant mt genomes [32, 36] or use of MAKER and Prokka software [35]. For identification of tRNAs, tRNAscan-SE, and ARAGORN were commonly used. To analyze repeats such programs as REPuter, RepeatMasker, RepeatModeler, and Tandem Repeat Finder were among most used. The combined machine learning and in silico enrichment of mitochondrial-like long reads were also used to assemble the mitogenome of Norway spruce [37].

Gene content reported for available annotated mitogenomes varies noticeably as well as the amount of repeats, showing no correlation between genome size, number of genes and repeats (Table 5).

**Table 5:** General features of plant mitogenomes

Species	GC, %	Genome size, Kbp	Protein coding genes	tRNA genes	Repeats, %	Reference
*Arabidopsis thaliana*	–	367	31	17	–	[47]
*Mielichhoferia elongata*	39.8	100	39	24	–	[48]
*Silene conica*	–	11,318	25	2	40.8	[5]
*Silene noctiflora*	–	6728	26	3	10.9	[5]
*Cycas taitungensis*	46.9	415	41	22	15.1	[32]
*Ginkgo biloba*	50.4	347	41	23	9.5	[33]
*Welwitschia mirabilis*	53.0	979	29	8	2.9	[33]
*Taxus cuspidata*	50.4	469	32	10	13.2	[34]
*Pinus taeda*	47.0	1191	41	12	14.2	(Table 1 in [34])
*Picea glauca*	44.7	5940	51	29	6.6	[35]
*Picea abies*	–	4300	41	–	–	[36]
*Picea abies*	44.7	4899	41	17	15.2	[37]
*Larix sibirica*	41.9	11,690	40	34	14.5	This study

Mitochondrial genomes often contain large amounts of plastid-derived DNA sequences (MTPTs). Analysis of the first gymnosperm mitochondrial genome of *Cycas taitungensis* and 10 other plants revealed that first DNA transfer from cpDNA to mtDNA occurred at least as far back as to the common ancestor of extant gymnosperms and angiosperms, about 300 mya [49]. Later, an extended analysis of MTPTs among 73 plant species was carried out, however, only one gymnosperm mitogenome was used there [50]. Mitogenome of Siberian larch also contained MTPTs. The longest MTPT is 10 Kbp long and contains the plastid genes *trnL*, *trnT*, *rps4*, *trnS*, *ycf3*, *psaA*, and *psaB* (Table 3). The discovered MTPTs would facilitate further studies of conifer mitogenomes and the understanding of organelle genome evolution.

The reasons for extremely large size of mitogenomes in plants are still not fully understood, but at least can be partly explained by a variable number of mobile genetic elements, introns and plasmid-related sequences [51] and could be affected by different factors, such as proliferation of retrotransposons, generation of repetitive DNA by recombination, and transferring of foreign sequences from plastid or nuclear genomes or via horizontal exchange of mitochondrial DNA (see [34] for discussion). However, it is unlikely that plasmids contributed much into the Siberian larch mitogenome size as we found relatively few plasmid-like insertions, representing only 0.11% of the entire mitogenome. Therefore, it seems that identified plasmids cannot explain much of that “dark matter” mtDNA.

As part of the genome annotation process, we inferred RNA editing sites for protein-coding genes. The number of RNA editing sites predicted by PREP-Mt for the protein-coding genes of the Siberian larch mitogenome was within the range predicted with the same cut-off 0.2 in other gymnosperms, but varied greatly between different species from 225 in *Welwitschia* to 1102 in *Taxus*, 1179 in *Pinus*, 1206 in *Cycas*, and 1306 in *Ginkgo* (see Table 1 in [34]. The number of RNA editing sites correlated neither with mitogenome size nor with GC content, but more data are needed to make stronger conclusions.

In the present study, we aimed to produce a high-quality assembly of the Siberian larch mitogenome. The combination of mtDNA enrichment with short and long reads allowed us to obtain a genome assembly, which consists of 9 contigs with a total length of 11.7 Mbp. Our assembly does not consist of one sequence, which is called the master ring, but many recent publications showed that presentation of plant mitogenome as a single circular molecule is not accurate due to its complexity and dynamic mixture of mtDNA forms within a single plant [52].

To facilitate mitogenome assembly, DNA was collected from a fraction enriched with mitochondria, however, such method did not allow the obtention of highly purified high molecular weight (HMW) mtDNA needed for long read sequencing. Moreover, the obtained nucleotide sequences contained a significant contamination from nuclear and chloroplast genomes. It appears that the protocol we used to isolate mtDNA from conifer needles would require further adjustments. Such improvements would likely focus on the damaging effect of larch phenolic compounds on mitochondrial membranes during the homogenization step, which would thus increase the amount of intact organelles. In turn, this should result in a larger yield of intact mtDNA molecules. To achieve this, it seems necessary to (i) shorten the mitochondrial isolation procedure as much as possible and (ii) find a better buffer composition for both extraction and washing media. Note that the use of fresh material instead of cryo-preserved one would also likely contribute to a better isolation procedure.

However, it is very important to notice that even with the best possible protocol for mtDNA enrichment and intact mtDNA molecules, it would still be very hard (if not currently impossible) to verify whether a particular large plant mitogenome assembly represents a single circular molecule or not. All subgenomic circles could theoretically be detected, if (and only if) extremely long reads comparable with the sizes of these subgenomic circles would be generated, which is still not feasible considering the huge length of conifer mitogenome. For unambiguous alternative assemblies reads should be as long as several Mbp. However, even though mitogenome would be assembled as a single circular structure, this would not guarantee its circular nature. Additionally, there could be alternative physical structures, which can all produce circular genome maps - such as head-to-tail concatamers and circularly permuted linear molecules (see Fig. 1 in [53]).

## Conclusions

Mitogenomes of conifer species are still poorly studied - only five genomes have been published to date. The sizes of published mitogenomes vary widely: from 986 Kbp in Scots pine (https://www.ncbi.nlm.nih.gov/assembly/GCA_900143225.1) to 11.7 Mbp in Siberian larch (this study). We succeeded in producing a high-quality assembly of the Siberian larch mitogenome using both short and long nucleotide sequencing reads generated by the Illumina HiSeq 2000 and MinION. The final assembly of the Siberian larch mitogenome consists of 9 contigs with a total length of 11,662,539 bp (N50 = 3,031,766 bp). The longest contig is 4,008,762 bp, the shortest - 24,767 bp. Finally, 40 protein-coding, 34 tRNA, and 3 rRNA genes were annotated. This mitogenome is currently the largest one among publicly known. The assembled genome is of sufficient quality for further detailed studies and comparative analyses with other plant mitogenomes.

## Methods

Mitogenome of Siberian larch was assembled from DNA sequences obtained from needles collected from a reference Siberian larch tree used in the de novo whole genome sequencing project [40] using two approaches. First, the total genomic DNA was isolated following mtDNA enrichment through isolation and purification of mitochondria. This DNA was used for sequencing on HiSeq 2000 platform (Illumina, Inc., San Diego, California, USA). Second, we isolated the HMW total genomic DNA to construct MP libraries for Illumina sequencing and to obtain long reads using MinION (Oxford Nanopore Technologies, Inc., Oxford, United Kingdom).

### MtDNA enrichment through isolation and purification of mitochondria

To enrich total DNA with mtDNA the following mitochondria isolation protocol was used. The Siberian larch needles were stored either at 4 °C for 4–13 days or at − 80 °C for 10–12 months after they were collected from a reference Siberian larch tree [40]. For long term storage, the needles were placed in a cryopreservation medium (10 mM MOPS, 5% DMSO, 5% glycerol, pH 7.4). The isolation procedure was based on the protocol reported in Sullivan et al. [37], with a few amendments as hereafter described. Before the mitochondria isolation procedure, the cryopreserved needles were thawed for 40–50 min by immersing them in warm water followed by the treatment for 1 min with 96% ethanol. Then, they were washed twice with distilled water. The weight of the plant material used was about 20 g. Further, the needles were cut into 1 cm pieces. Needles were homogenized in portions of 7 g each in 50 ml of chilled extraction medium (EM) containing 0.35 M mannitol, 30 mM MOPS, 1.25 mM EGTA, 2 mM sodium metabisulfite, 2.5 mM MgCl_2_, 0.2% (w/v) BSA, 0.3% (w/v) PVP-40, 0.3% (w/v) PVPP, 3 mM DTT, 3 mM cysteine, pH 7.4. Homogenization was carried out in a blender at 4 °C in series of 9 times for 3 s each and 3 times for 10 s each, followed by the grinding in a cooled mortar using pestle for 50–60 s. The homogenate was filtered through 4 layers of gauze and 2 layers of 50 μm nylon gauze. Then, 50 ml of EM were added to the homogenate while filtering (the total weight ratio of the needles to EM volume at the end of the homogenization was 1:16). The homogenate was centrifuged at 2500 g in F0685 Beckman Rotor for 5 min. The supernatant was centrifuged at 5000 g for 10 min. The pellet was discarded. The supernatant was centrifuged again at 13,000 g for 15 min. The pellet was resuspended in 100 ml of the washing medium (WM): 0.3 M mannitol, 30 mM MOPS, 0.2% (w/v) BSA, pH 7.4. The resulting suspension was centrifuged at 13,000 g for 15 min. The pellet was resuspended in 25 ml of the WM. The suspension was then centrifuged at 13,000 g for 15 min. The pellet was resuspended in 1 ml of the WM. The resulting suspension was centrifuged at 11,000 g for 10 min (HL 081 F45–24-11 Rotor) using the 5415R centrifuge (Eppendorf AG, Hamburg, Germany). The final mitochondrial pellet was resuspended in the WM without BSA in a 1: 1 weight/volume ratio.

### MtDNA extraction from isolated mitochondria

To isolate mtDNA, 3 μl of DNase from Sigma-Aldrich (St. Louis, Missouri, USA) (1 mg/1 ml) and 3 μl of 1 M MgCl_2_ were added to 200 μl of the final mitochondrial suspension. The mixture was incubated for 20 min at 25 °C. Then, 1 ml of 0.3 M mannitol, 30 mM MOPS, 10 mM EDTA, 10 mM EGTA, pH 7.4 was added. The resulting mixture was centrifuged at 11,000 g for 5 min. The pellet was resuspended in 1 ml of 0.3 M mannitol, 30 mM MOPS, pH 7.4 and centrifuged again at 11,000 g for 5 min. Then, DNA was isolated from the resulting pellet using DNeasy Plant Mini Kit from QIAGEN (Hilden, Germany) according to the manufacturer’s protocol.

### DNA isolation for long reads

The isolation of high-quality HMW DNA for further sequencing on MinION was carried out using the modified CTAB-method [54]. Fresh needles (0.05–0.07 g per tube) were placed in 700 μL pre-warmed (65 °C) 2% CTAB isolation buffer (2% CTAB 1.4 M NaCl, 100 mM Tris pH 8.0, 20 mM EDTA) with the addition of 120 μl Proteinase K from Bioron GmbH (Römerberg, Germany). The mixture was transferred to a 1.5-mL microcentrifuge tube and incubated at 65 °C for 2 h. Then, 4 μl RNase was added to the mixture and incubated for 30 min at 65 °C. DNA was purified once with phenol-chloroform (1:1) and twice with one volume chloroform and then DNA was precipitated with one volume isopropanol. The obtained pellet was washed with 70% EtOH, dried, and dissolved in 50 μL TE buffer. Then, DNA solution from 12 tubes was combined in a single tube and concentrated in 100 μL TE buffer using AMPure XP beads (Beckman Coulter Inc., Brea, CA, USA) at 2:1 ratio. Resulting DNA solution was run on a 1% low-melting temperature agarose gel in 0.1% TAE buffer, and a HMW fraction was excised with a sterile razor blade, placed in a microcentrifuge tube, frozen at − 70 °C, and then melted. The frosting-melting cycle was repeated three times, and after the third thawing, the tube was centrifuged, and the aqueous layer was transferred to a new tube. Then, phenol-chloroform purification and precipitation with isopropanol was performed one again. DNA purity was examined with absorbance ratios using NanoPhotometer P300 Spectrophotometer (Implen GmbH, München, Germany), and concentration was measured using Qubit Fluorimeter (Thermo Fisher Scientific Inc., Waltham, Massachusetts, USA). Finally, the Ligation Sequencing kit 1D (SQK-LSK108) was used to prepare library for the Nanopore sequencing. Elution of DNA from agarose gel was necessary to reduce contaminants co-purification together with DNA from larch needle samples. Skipping this step always led to very fast (in 15–20 min) dying of the population of nanopores on a flow cell.

### DNA sequencing

All sequencing was performed in the Laboratory of Forest Genomics (Genome Research and Education Center, Siberian Federal University, Krasnoyarsk, Russia). The PE library with the mean insertion size of 700 bp was prepared from the enriched mtDNA using the Illumina TruSeq DNA LT Sample Prep Kit according to the Illumina TruSeq DNA Sample Preparation Guide (Illumina Inc., San Diego, CA). Three MP libraries were prepared from the total non-enriched DNA using Nextera Mate Pair Library Prep Kit (Illumina Inc., San Diego, CA). These PE and 3 MP libraries were sequenced with 2 × 100 cycles on the HiSeq 2000 platform using the Illumina TruSeq SBS Kit v3 (Illumina Inc., San Diego, CA). More detailed data on Illumina libraries and obtained reads are provided in Table 6.

**Table 6:** Data on type and size of Illumina libraries used for sequencing and obtained reads

Library type	Number of read pairs	Total length, Gbp	mtDNA enrichment	Insert size, Kbp
MP	143,824,061	23.9	No	2–3
MP	245,866,919	38.7	No	5–7
MP	235,758,577	38.2	No	8–10
PE	19,680,530	4.1	Yes	0.7

The total non-enriched DNA library was sequenced on the MinION (Oxford Nanopore Technologies Inc., Oxford, United Kingdom) with use of R9 FlowCells (FLO-MIN106).

### RNA isolation, sequencing and assembly

RNA was isolated from autumn buds of a reference Siberian larch tree [40] using the Qiagen RNeasy Mini Kit (Qiagen, Hilden, Germany). The RNA-seq library was prepared using the TruSeq RNA Sample Preparation Kit v2 (Illumina Inc., San Diego, CA). In brief, mRNA was purified and fragmented followed by cDNA synthesis. Then, end repair, adapter ligation, and size selection using AMPure XP beads were done. The cDNA was PCR amplified. The sequencing of the obtained library was carried out on the MiSeq platform with the Illumina MiSeq Reagent Kit v2 (2 × 150-cycles) (Illumina Inc., San Diego, CA). The RNA reads were trimmed using Phred quality score 30 and base call accuracy 99.9% using FastQC v. 0.11.5 (http://www.bioinformatics.babraham.ac.uk/projects/fastqc). The de novo transcriptome assembly was generated using Trinity v. 2.8.4 (https://github.com/trinityrnaseq/trinityrnaseq/wiki).

### Preliminary assembly using Illumina PE and MP short reads

The quality of Illumina reads was assessed using FastQC v. 0.11.5 [55]. Adapter sequences were trimmed and short reads were filtered using Trimmomatic v. 0.36 [56] with minimum quality of 19 and minimum length of 35 bp. Data on the PE and MP libraries and sequencing reads are presented in Table 6.

The obtained PE and MP sequence reads were assembled de novo into contigs using the CLC Assembly Cell v. 5.0.0 [57]. Scaffolding was performed using BESST [58]. Gap closing was done using Sealer (https://github.com/bcgsc/abyss/tree/master/Sealer). The resulting assembly of Illumina reads consisted of 1,216,421 contigs with a total length of 516,8 Mbp, N50 = 436 bp and the longest contig 1,449,395 bp.

### Hybrid assembly using both Illumina short PE and MinION long reads

The basecalling and the quality evaluation for MinION reads were performed using Albacore [59]. After trimming and filtering, the average read length was 4523 bp, the average quality was 9.1, the total length - 6.2 Gbp, and the longest read - 77,840 bp.

The hybrid assembly using long MinION reads and short PE Illumina reads was carried out using MaSuRCA v. 3.2.8 [60]. This hybrid assembly consisted of 10,909 contigs with a total length of 55,3 Mbp, N50 = 7516 bp and the longest contig 4,008,762 bp.

To mine mitochondrial contigs from this assembly, the BLAST search against all mitochondrial plant sequences available in the NCBI GenBank was used. After mapping this assembly to plant mitochondrial database, we finally assembled 9 mitochondrial contigs with total length of 11.7 Mbp. The largest contig was 4,008,762 bp long.

### Hybrid assembly evaluation

To further evaluate the accuracy of the hybrid assembly we ran REAPR v1.0.18 [41]. It uses paired reads mapping information to search for low or exceedingly high coverage regions, mis-orientation of read pairs, high local SNP densities, and correlated SNPs to indicate mis-assemblies and collapsed repeats. The program scans for the four types of error: a region with or without a gap that triggered an FCD error and a region with low fragment coverage that does or does not contain a gap.

### Mitogenome annotation

Mitogenome of Siberian larch was checked for homology with other plant mitogenomes existing in the NCBI GenBank database using the NCBI BLAST algorithm. Mitofy [16] was also used for Siberian larch mitogenome annotation, but only 11 genes were found with this tool. The tRNA genes were discovered using ARAGORN [61] and tRNAscan-SE [62] tools. Ribosomal RNA (rRNA) were annotated using RNAmmer [63].

RNA editing sites were predicted using PREP-Mt [64] with a cutoff value of 0.2. They were verified by aligning RNA-seq reads to the annotated mitochondrial genes and calling variants with the CLC Genomic Workbench.

To search for MTPTs we used CLC Genomics Workbench whole genome alignment tool and Siberian larch chloroplast genome sequence [46].

### Repetitive element (RE) analysis

RepeatModeler v.1.0.11 [65] was used to search for REs in the mitogenome assembly. TEclass online service [66] was employed to classify unknown repeated elements from de novo RE library generated by RepeatModeler. In addition to this RepeatModeler derived library and RepBase library [67], MIPS Repeat Element Database library [68], Custom Plant Repeat Database [69], and Pine Interspersed Repeats Resource library PIER v1.0 [38, 70] were used to generate a combined repeat library to run RepeatMasker v. 4.0.6 [70]. Custom R script was applied to parse RepeatMasker results according to RepBase classification [68].

### Search for plasmids

To test whether the Siberian larch mitogenome contains plasmid-like sequences all nine contigs assembled in this study were blasted against 1) a linear plasmid sequence identified in mitochondria of *Picea abies* (L.) Karst and deposited at the NCBI GenBank under accession number AJ225562 and 2) 891 broad host range plasmids (NCBI GenBank taxid:36549). The blast was performed with different stringency searching for highly similar sequences (megablast), more dissimilar sequences (discontiguous megablast), and somewhat similar sequences (blastn).

## Supplementary information

**Additional file 1.** The Siberian larch mitogenome assembly evaluation using REAPR v1.0.18.

**Additional file 2.** Plasmids matching inserts in the nine Siberian larch mitogenome contigs and their NCBI GenBank accession numbers.

## Data Availability

All sequences for nine Siberian larch mitochondrial contigs described in this study are available in the NCBI GenBank under the accession numbers MT797187-MT797195.

## Abbreviations

ADP: Adenosine 5′-diphosphate
blast: Basic local alignment search tool
BSA: Bovine serum albumin
*bp*: Base pair
CCCP: Carbonyl cyanide 3-chlorophenylhydrazone
cpDNA: Cloroplast DNA
DMSO: Dimethyl sulfoxide
DNase: Deoxyribonuclease
DTT: Threo-1,4-Dimercapto-2,3-butanediol or DL-Dithiothreitol
EGTA: Ethylene-bis (oxyethylenenitrilo) tetraacetic acid)
EDTA: Ethylenediaminetetraacetic acid
g: Gram
HMW: High molecular weight
Kbp: Kilo base pair
*LINE*: Long interspersed nuclear element
LTR: Long terminal repeat
MOPS: 3-(N-Morpholino) propanesulfonic acid
MP: Mate-pair
mtDNA: Mitochondrial DNA
mya: Million years ago
PE: Paired-end
PVP: Polyvinylpyrrolidone
PVPP: Polyvinylpyrrolidone, cross-linked
ORF: Open reading frame
rRNA: Ribosomal RNA
*SINE*: Interspersed nuclear element
tRNA: Transfer RNA
μl: Microliter
MTPT: Mitochondrial plastid DNA

## Gene abbreviations

*atp1*, *atp4*, *atp6*, *atp8* and *atp9*: Genes for ATP synthase subunits 1, 4, 6, 8 and 9
*ccmC*, *ccmC*, *ccmFc* and *ccmFn*: Genes for *cytochrome c biogenesis proteins B, C, FC and FN*
*cob*: Gene for cytochrome b
*cox1, cox2* and *cox3*: Genes for cytochrome c oxidase subunits 1, 2 and 3
*matR*: *Gene for maturase*
*mttB*: transport membrane protein B
*nad1, nad2, nad3, nad4, nad4L, nad5*, *nad6, nad7 and nad9*: Mitochondrial genes for NADH dehydrogenase subunits 1–7, 9 and 4 L
*psaA*: gene for photosystem I P700 apoprotein A1
*psaB*: gene for photosystem I P700 apoprotein A2
*rpl2, rpl5 and rpl16*: *Genes for ribosomal proteins L2, L5 and L16*
*rps1, rps2, rps3, rps4, rps7, rps10, rps11, rps12, rps13, rps14 and rps19*: *Genes for ribosomal proteins S1, S2, S3, S4, S7, S10, S11, S12, S13, S14, and S19*
*sdh3 and sdh4*: *Genes for succinate dehydrogenase cytochrome subunits 3 and 4*
*trnL*: tRNA gene for leucine
*trnT*: tRNA gene for threonine
*trnS*: tRNA gene for serine
*ycf3*: gene for photosystem I assembly protein Ycf3
