# Supplementary material for: Siberian larch (Larix sibirica Ledeb.) mitochondrial genome assembled using both short and long nucleotide sequence reads is currently the largest known mitogenome
Source: BMC Genomics. 2020 Sep 23;21:654. doi: 10.1186/s12864-020-07061-4 (PMC7517811; doi:10.1186/s12864-020-07061-4)
Supplement: Supplementary file 1 — Additional file 1. The Siberian larch mitogenome assembly evaluation using REAPR v1.0.18. [file 12864_2020_7061_MOESM1_ESM.pdf]

The Siberian larch mitogenome assembly evaluation using REAPR v1.0.18

First, using REAPR GC-coverage bias and fragment size distribution were estimated. Fig. S1 demonstrates the expected insert size distribution in the assembly based on PE reads alignment.

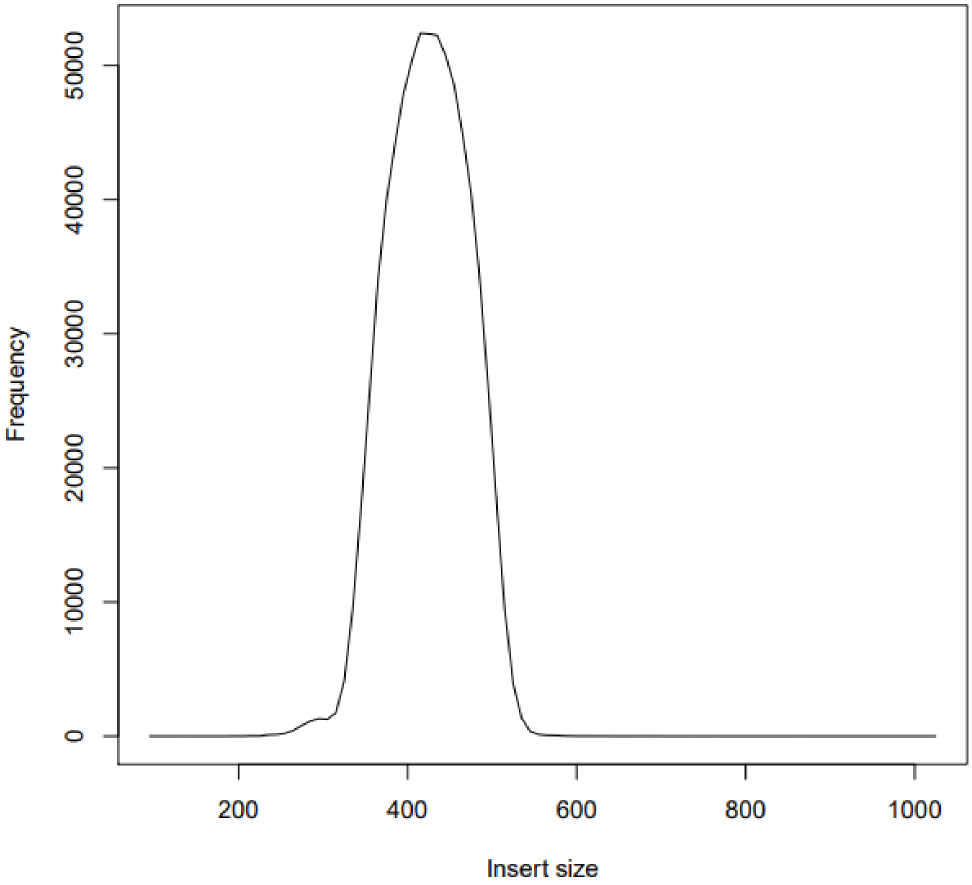

Fig. S1 Expected insert size distribution in the assembly based on PE reads alignment

Then, REAPR used paired read mapping information to scan for the four types of error: a region with or without a gap that would trigger an FCD error (Fig. S2) and a region with low fragment coverage that does or does not contain a gap (Fig. S3).

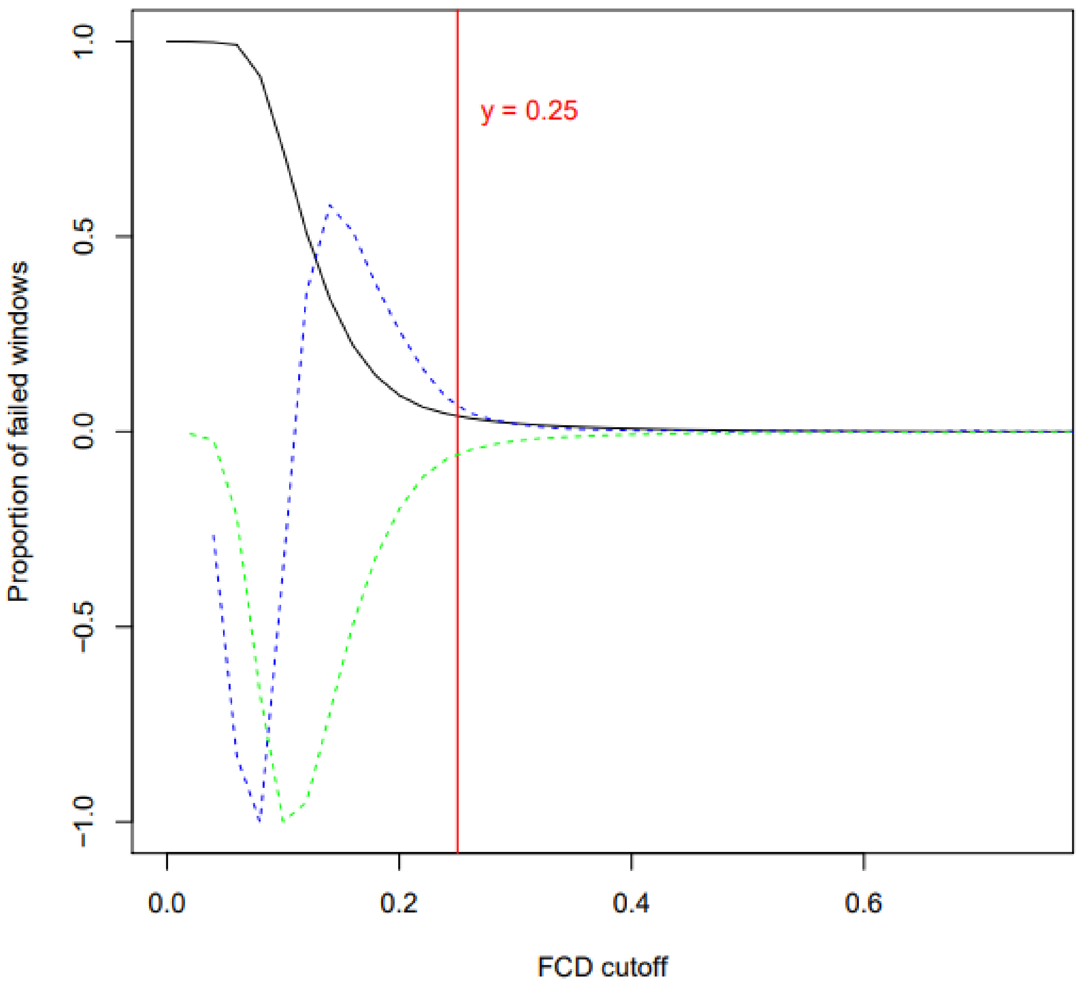

Fig. S2 Calculated fragment coverage distribution (FCD) error cut-off. The black line shows the proportion of 100-bp windows that would fail and hence be labelled as an assembly error at each cutoff value. The dashed green and blue lines are the first and second derivatives of the black line, respectively. The red line shows the chosen cutoff value

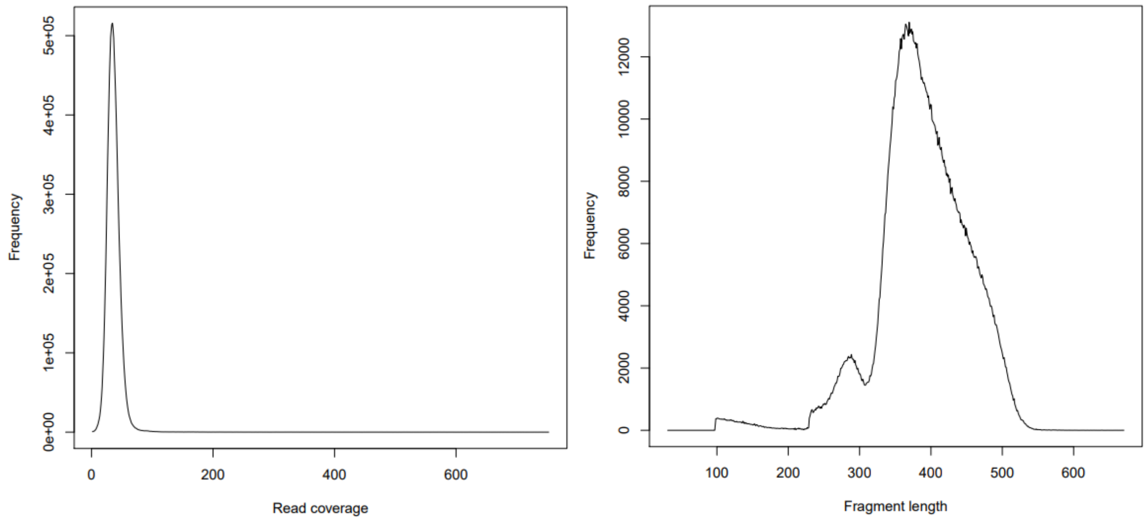

Fig. S3 Distribution of read coverage (left) and fragment distribution (right) in the assembly of Siberian larch mitogenome

For the Siberian larch (*Larix sibirica*) mitogenome assembly REAPER reported 92.13% of error free bases, which is comparable to 86% for the GRCh37 human reference genome or 90.3% for *C. elegans* genome (Hunt et al. 2013) [40]. Two scaffolds were broken according to the estimated FCD error threshold, which means the errors occurred at the gapped regions, as REAPR breaks assembly only if the error contains a gap. Therefore, as a result, from initial seven scaffolds nine contigs were finally generated and presented.

Reference

Hunt M, Kikuchi T, Sanders M, Newbold C, Berriman M, Otto TD. REAPR: a universal tool for genome assembly evaluation. Genome Biol. 2013;14(5):R47.
